# Factors Affecting Use and Delay (≥8 Weeks) of Adjuvant Chemotherapy after Colorectal Cancer Surgery and the Impact of Chemotherapy-Use and Delay on Oncologic Outcomes

**DOI:** 10.1371/journal.pone.0138720

**Published:** 2015-09-18

**Authors:** Ik Yong Kim, Bo Ra Kim, Young Wan Kim

**Affiliations:** 1 Department of Surgery, Division of Colorectal Surgery, Yonsei University Wonju College of Medicine, Wonju, Korea; 2 Department of Internal Medicine, Division of Gastroenterology, Yonsei University Wonju College of Medicine, Wonju, Korea; University of Algarve, PORTUGAL

## Abstract

**Purpose:**

To evaluate factors affecting the use and delay ≥8 weeks of adjuvant chemotherapy and the impact of chemotherapy use and delay on survival.

**Methods:**

Between 2005 and 2012, consecutive patients with stage II and III colorectal cancer who were treated with major curative resection were enrolled.

**Results:**

Among 750 patients with stage II (n = 318) and III (n = 432) disease, 153 (20.4%) did not receive chemotherapy. Among 597 patients with adjuvant chemotherapy, 31 (5.2%) began chemotherapy 8 weeks or more after surgery. Factors associated with not receiving chemotherapy were: age ≥80 years (hazard ratio [HR] = 5.2), American Society of Anesthesiologists score ≥3 (HR = 1.9), underlying cerebrovascular disease (HR = 1.7), stage II disease (HR = 2.0), presence of postoperative complications (HR = 2.2), or intensive care unit admission (HR = 2.4). Factors associated with chemotherapy delay ≥8 weeks were: male sex (HR = 4.2), rectal primary cancer (HR = 5.4), or presence of postoperative complications (HR = 2.5). Independent prognostic factors for overall survival included TNM III stage (HR = 2.04) and chemotherapy delay ≥8 weeks (HR = 0.39) or <8 weeks (HR = 0.22). Independent prognostic factors for recurrence-free survival were TNM III stage (HR = 2.26) and chemotherapy delay <8 weeks (HR = 0.35).

**Conclusions:**

Postoperative complications were associated with both lack of and delayed chemotherapy. Timely initiation of chemotherapy, defined as <8 weeks, was a favorable prognostic factor for overall and recurrence-free survival. To increase the proportion of patients receiving chemotherapy and timely initiation of chemotherapy, surgical complications should be minimized after curative resection.

## Introduction

Curative surgical resection is the primary treatment modality for colorectal cancer. After resection, adjuvant chemotherapy is performed to reduce the risk of metastasis and recurrence [1]. Although quantifying the oncologic benefits of adjuvant chemotherapy is difficult, chemotherapy with fluorouracil and folinic acid improves survival by 3.6% in patients with stage II colorectal cancer [2]. According to a meta-analysis by Dube *et al*.[3], adjuvant chemotherapy with fluorouracil improved survival by 5% for patients with Dukes C colon cancer and adjuvant chemoradiation therapy increased survival by 9% for patients with Dukes B and C rectal cancer. In 2011, only 64% of patients with stage III colon cancer received adjuvant chemotherapy in the United States [4]. Use of chemotherapy is associated with age, race, underlying disease, marital and economic status, and occurrence of postoperative complications [5].

The impact of delayed chemotherapy on survival is controversial because of the paucity of high-level evidence. Factors associated with chemotherapy delay are age, race, postoperative recovery, underlying disease, histologic grade, and marital status [6].

Although current National Comprehensive Cancer Network (NCCN) guidelines recommend adjuvant chemotherapy for people with stage II and III colorectal cancer after surgery [7, 8], no guidelines have been established for the time to chemotherapy initiation. Most clinical trials favor initiation of adjuvant chemotherapy for colon cancer within 6 to 8 weeks after surgery [9, 10]. Thus, a randomized trial to evaluate the impact of delayed chemotherapy on patient survival would be unethical.

To date, the reasons for use and delay of adjuvant chemotherapy have not been extensively studied and the association between chemotherapy delay and oncologic outcomes remains unclear. This study aimed to evaluate factors affecting the use and delay ≥8 weeks of chemotherapy after colorectal cancer surgery and the impact of chemotherapy use and delay on oncologic outcomes.

## Methods

### Patients

This retrospective cohort study was performed at a tertiary referral center following the Strengthening the Reporting of Observational Studies in Epidemiology (STROBE) guidelines[11]. All clinical investigations were conducted according to the principles expressed in the Declaration of Helsinki and were approved by the Institutional Review Board of Wonju Severance Christian Hospital (YWMR-14-5-099). All participants provided their written informed consent to participate in this study and the ethics committee approved this consent procedure. Informed consent was obtained from all patients before surgery. Between 2005 and 2012, 750 consecutive patients undergoing major resection for stage II and III colorectal cancer were enrolled. Eligibility criteria were histologically confirmed colorectal cancer and major colorectal resection with curative intent. Patients with stage I and IV disease and those undergoing R2 resection for macroscopic residual disease or nonresectional procedures for colorectal cancer were excluded.

### Study endpoints

The primary endpoint was identifying factors affecting use and delay ≥8 weeks of adjuvant chemotherapy after colorectal cancer surgery. The secondary endpoint was the impact of use and delay of chemotherapy ≥8 weeks on oncologic outcomes.

### Preoperative chemoradiation therapy

Patients with clinical stage T3 or T4 and/or node-positive rectal cancer underwent preoperative chemoradiation therapy. A long course schedule was used and total radiation dose was 50.4 Gy. Radiation was delivered to the entire pelvis (45 Gy in 25 fractions) with a boost to the primary tumor (5.4 Gy in 3 fractions) over 5 weeks. Intravenous chemotherapy (425 mg/m^2^ 5-fluorouracil and 20 mg/m^2^ leucovorin) was administered during weeks 1 and 5 of radiation therapy.

### Surgery, adjuvant therapy, and follow-up

After standardized preoperative preparations, standard surgical procedures were performed. Complete mesocolic excision for colon cancer and total mesorectal excision for rectal cancer were performed using standard surgical procedures.

After recovery from surgery, all patients with stage II and III disease were recommended to receive chemotherapy according to NCCN guidelines. All cases were discussed at weekly multidisciplinary team meetings. Oxaliplatin or irinotecan-containing regimens were considered for stage II patients with high-risk features (tumors that were T4 or histological grade 3 or 4; peritumoral lymphovascular involvement; bowel obstruction; T3 lesions with localized perforation; positive resection margin; or perineural invasion). Chemotherapy regimens included fluoropyrimidine (fluorouracil with folinic acid and capecitabine) alone or in combination with oxaliplatin (FOLFOX)/irinotecan (FOLFIRI).

Adjuvant radiation was used for patients with stage II and III rectal cancer. Adjuvant radiation therapy was performed as follows. Fluorouracil-based chemotherapy consisted of 425 mg/m^2^ of 5-fluorouracil and 20 mg/m^2^ of leucovorin for 5 days every 28 days for six cycles. Radiation therapy was performed after the second round of chemotherapy and consisted of 50.4 to 54 Gy delivered in 28 to 30 fractions.

All surgical patients were registered in a dedicated colorectal database and followed at 3- or 6-month intervals for the first 5 years and then yearly thereafter.

### Outcome measures

Time to chemotherapy was defined as the duration from surgery to initiation of adjuvant chemotherapy. Chemotherapy delay was defined using 8 weeks as the cutoff. Postoperative complications were defined as events that required additional treatment within 30 days of surgery, based on the Clavien-Dindo classification. Conversion to open surgery was defined as completion of planned surgical procedures using a conventional laparotomy incision. Treatment-related variables such as intensive care unit (ICU) care or blood transfusions were included in analysis if they were required within 48 hours after primary surgery. The 48-hour limit sought to assess intraoperative patient burdens rather than postoperative complications. We assumed that ICU admission or transfusion within 48 hours indicated immediate surgical stress.

### Statistical analysis

All statistical analyses used MedCalc Statistical Software version 15.2.2 (MedCalc Software bvba, Ostend, Belgium) and IBM SPSS Statistics for Windows, version 21.0 (IBM, Armonk, NY, USA). Categorical variables were described by frequencies and percentages and compared using chi-square test or Fisher's exact test as appropriate. Continuous variables were described as mean and standard deviation and analyzed by Student’s *t*-test. Factors associated with use and delay of chemotherapy were identified by logistic regression analysis. Survival analysis was by the Kaplan-Meier method with log rank tests and the Cox proportional hazard model. A *p*-value <0.05 was considered statistically significant.

## Results

### Details of adjuvant chemotherapy

Among 750 patients with stage II (n = 318) or III (n = 432) disease, adjuvant chemotherapy was performed in 597 (79.6%). Among 597 patients with adjuvant chemotherapy, 31 (5.2%) received chemotherapy 8 weeks or more after surgery. Detailed chemotherapy data are presented in **Table 1**.

**Table 1:** Adjuvant chemotherapy.

			TNM stage		
			II (n = 318)	III (n = 432)	
			N (%)	N (%)	P
Chemotherapy use	(-) (n = 153)		83 (26.1)	70 (16.2)	0.001
	(+) (n = 597)		235 (73.9)	362 (83.8)	
Time to chemotherapy	<8 weeks (n = 566)	Fluoropyrimidine	182 (82.4)	187 (54.2)	<0.001
		Oxaliplatin	38 (17.2)	149 (43.2)	
		Irinotecan	1 (0.5)	9 (2.6)	
	≥8 weeks (n = 31)	Fluoropyrimidine	11 (78.6)	15 (88.2)	0.717
		Oxaliplatin	1 (7.1)	1 (5.9)	
		Irinotecan	2 (14.3)	1 (5.9)	

### Factors associated with no use of adjuvant chemotherapy

Based on multivariate analysis, factors associated with not receiving chemotherapy were age ≥80 years (hazard ratio [HR] = 5.2), American Society of Anesthesiologists (ASA) score ≥3 (HR = 1.9), underlying cerebrovascular disease (HR = 1.7), stage II disease (HR = 2.0), presence of postoperative complications (HR = 2.2) and ICU admission (HR = 2.4). The use of preoperative chemoradiation for rectal cancer increased the use of adjuvant chemotherapy (11.4% vs. 4.6%, p = 0.012). However, in multivariate analysis, this was not an independent factor for the use of chemotherapy (**Table 2**).

**Table 2:** Factors associated with not receiving adjuvant chemotherapy (n = 750).

		Univariate analysis			Multivariate analysis	
		Chemotherapy (-)	Chemotherapy (+)	P	HR (95% CI)	P
		N (%)	N (%)			
Age (years)	<80	97 (63.4)	549 (92)	<0.001	1	<0.001
	≥80	56 (36.6)	48 (8)		5.2 (3.2–8.4)	
Age subgroups	≤70	39 (9.9)	356 (90.1)	<0.001	NA	
	71–75	34 (19.8)	138 (80.2)			
	76–80	35 (33.3)	70 (66.7)			
	81–85	27 (50.9)	26 (49.1)			
	≥86	18 (72)	7 (28)			
Sex	Male	93 (60.8)	382 (64)	0.463	NA	
	Female	60 (39.2)	215 (36)			
BMI	≥30 kg/m^2^	9 (5.9)	18 (3)	0.089	NA	
ASA score	3,4	48 (31.4)	81 (13.6)	<0.001	1.9 (1.1–3.1)	0.014
Comorbidity						
Diabetes		36 (23.5)	112 (18.8)	0.186	NA	
Cardiovascular disease		81 (52.9)	254 (42.5)	0.021	1.2 (0.8–1.8)	0.479
Pulmonary disease		18 (11.8)	62 (10.4)	0.622	NA	
Kidney disease		5 (3.3)	7 (1.2)	0.065	NA	
Liver disease		4 (2.6)	19 (3.2)	0.716	NA	
Cerebrovascular disease		43 (28.1)	89 (14.9)	<0.001	1.7 (1.1–2.8)	0.026
Emergency	(+)	7 (5.6)	24 (5.2)	0.850	NA	
T4 tumor	(+)	27 (17.6)	143 (24)	0.096	NA	
National health insurance	Medical care	22 (14.4)	59 (9.9)	0.110	NA	
Region	Rural (*vs*. urban)	35 (22.9)	167 (28)	0.205	NA	
Preoperative CRT	(+)	7 (4.6)	68 (11.4)	0.012	0.5 (0.2–1.2)	0.105
Location	Colon	87 (56.9)	324 (54.3)	0.566	NA	
	Rectum	66 (43.1)	273 (45.7)			
Surgical approach	Open	70 (45.8)	243 (40.7)	0.259	NA	
	Minimally invasive	83 (54.2)	354 (59.3)			
Name of operation	APR, Hartmann	24 (15.7)	62 (10.4)	0.445	NA	
	LAR	44 (28.8)	217 (36.3)			
	PC	1 (0.7)	5 (0.8)			
	AR, LHC	42 (27.5)	154 (25.8)			
	TC, STC	4 (2.6)	10 (1.7)			
	RHC	37 (24.2)	144 (24.1)			
	Other	1 (0.7)	5 (0.8)			
TNM	II	83 (54.2)	235 (39.4)	0.001	2.0 (1.4–3.1)	0.001
	III	70 (45.8)	362 (60.6)		1	
Metastatic lymph node (number)	1–3	43 (61.4)	235 (64.9)	0.577	NA	
	≤4	27 (38.6)	127 (35.1)			
CRM	≤1 mm	10 (15.2)	50 (18.3)	0.546	NA	
Histologic grade	G3, G4	8 (5.2)	51 (8.5)	0.174	NA	
30-day complications	(+)	73 (47.7)	170 (28.5)	<0.001	2.2 (1.5–3.3)	<0.001
Anastomotic leakage	(+)	11 (7.2)	24 (4)	0.097	NA	
Clavien-Dindo grade	3, 4, 5	39 (25.5)	72 (12.1)	<0.001	NA	
ICU admission	(+)	82 (53.6)	148 (24.8)	<0.001	2.4 (1.6–3.7)	<0.001
Transfusion	(+)	62 (40.5)	178 (29.8)	0.011	1.1 (0.7–1.8)	0.563
Operative time	≥300 min	9 (7.3)	52 (11.3)	0.191	NA	
Open conversion	(+)	11 (13.3)	35 (9.9)	0.368	NA	

BMI, body mass index; ASA, American Society of Anesthesiologists; CRT, chemoradiation; APR, abdominoperineal resection; LAR, low anterior resection; PC, proctocolectomy; AR, anterior resection; LHC, left hemicolectomy; TC, total colectomy; STC, subtotal colectomy; RHC, right hemicolectomy; TNM, tumor-node-metastasis; CRM, circumferential resection margin; ICU, intensive care unit; HR, hazard ratio; CI, confidence interval; NA, not applied

### Factors associated with adjuvant chemotherapy delay

Based on multivariate analysis, factors affecting chemotherapy delay ≥8 weeks were male sex (HR = 4.2), rectal primary cancer (HR = 5.4), and the presence of postoperative complications (HR = 2.5). The use of preoperative chemoradiation treatment for rectal cancer did not influence adjuvant chemotherapy delay. Rates of adjuvant chemotherapy were 19.4% in patients with 8 or more weeks and 11% in patients who received treatment in less than 8 weeks (**Table 3**).

**Table 3:** Factors associated with a ≥8-week delay to adjuvant chemotherapy (n = 597).

		Univariate			Multivariate	
		≥8 weeks	<8 weeks	P	HR (95%CI)	P
		N (%)	N (%)			
Age (years)	<80	29 (93.5)	520 (91.9)	0.738	NA	
	≥80	2 (6.5)	46 (8.1)			
Age subgroups	≤70	19 (5.3)	337 (94.7)		NA	
	71–75	9 (6.5)	129 (93.5)			
	76–80	2 (2.9)	68 (97.1)			
	81–85	1 (3.8)	25 (96.2)			
	≥86	0 (0)	7 (100)			
Sex	Female	3 (9.7)	212 (37.5)	0.002	1	0.022
	Male	28 (90.3)	354 (62.5)		4.2 (1.2–14.2)	
BMI	≥30 kg/m^2^	0 (0)	18 (3.2)	0.313	NA	
ASA score	3,4	5 (16.1)	76 (13.4)	0.669	NA	
Comorbidity						
Diabetes		6 (19.4)	106 (18.7)	0.931	NA	
Cardiovascular disease		12 (38.7)	242 (42.8)	0.657	NA	
Pulmonary disease		5 (16.1)	57 (10.1)	0.282	NA	
Kidney disease		0 (0)	7 (1.2)	0.533	NA	
Liver disease		2 (6.5)	17 (3)	0.287	NA	
Cerebrovascular disease		8 (25.8)	81 (14.3)	0.08	NA	
Emergency	(+)	1 (4.5)	23 (5.3)	0.885	NA	
T4 tumor	(+)	11 (35.5)	132 (23.3)	0.122	NA	
National health insurance	Medical care	4 (12.9)	55 (9.7)	0.563	NA	
Region	Rural (*vs*. urban)	10 (32.3)	157 (27.7)	0.585	NA	
Preoperative CRT	(+)	6 (19.4)	62 (11)	0.152	NA	
Location	Colon	5 (16.1)	319 (56.4)	<0.001	1	0.001
	Rectum	26 (83.9)	247 (43.6)		5.4(2–14.5)	
Surgical approach	Open	17 (54.8)	226 (39.9)	0.1	NA	
	Minimally invasive	14 (45.2)	340 (60.1)			
Name of operation	APR, Hartmann	5 (16.1)	57 (10.1)	0.001	NA	
	LAR	22 (71)	195 (34.5)			
	PC	0 (0)	5 (0.9)			
	AR, LHC	2 (6.5)	152 (26.9)			
	TC, STC	0 (0)	10 (1.8)			
	RHC	2 (6.5)	142 (25.1)			
	Other	0 (0)	5 (0.9)			
TNM	II	14 (45.2)	221 (39)	0.497	NA	
	III	17 (54.8)	345 (61)			
Metastatic lymph node (number)	1–3	10 (65.2)	225 (58.8)	0.590	NA	
	≤4	7 (34.8)	120 (41.2)			
CRM	≤1 mm	5 (19.2)	45 (18.2)	0.899	NA	
Histologic grade	G3, G4	0 (0)	51 (9)	0.081	NA	
30-day complications	(+)	18 (58.1)	152 (26.9)	<0.001	2.5 (1.1–5.4)	0.021
Anastomotic leakage	(+)	8 (25.8)	16 (2.8)	<0.001	NA	
Clavien-Dindo grade	3, 4, 5	12 (38.7)	60 (10.6)	<0.001	NA	
ICU admission	(+)	8 (25.8)	140 (24.7)	0.893	NA	
Transfusion	(+)	15 (48.4)	163 (28.8)	0.02	2 (0.9–4.3)	0.077
Operative time	≥300 min	3 (13.6)	49 (11.2)	0.723	NA	
Open conversion	(+)	5 (35.7)	30 (8.8)	0.001	NA	

BMI, body mass index; ASA, American Society of Anesthesiologists; CRT, chemoradiation; APR, abdominoperineal resection; LAR, low anterior resection; PC, proctocolectomy; AR, anterior resection; LHC, left hemicolectomy; TC, total colectomy; STC, subtotal colectomy; RHC, right hemicolectomy; TNM, tumor-node-metastasis; CRM, circumferential resection margin; ICU, intensive care unit; HR, hazard ratio; CI, confidence interval; NA, not applied

### Oncologic outcomes by use and delay of adjuvant chemotherapy

**Fig 1:**
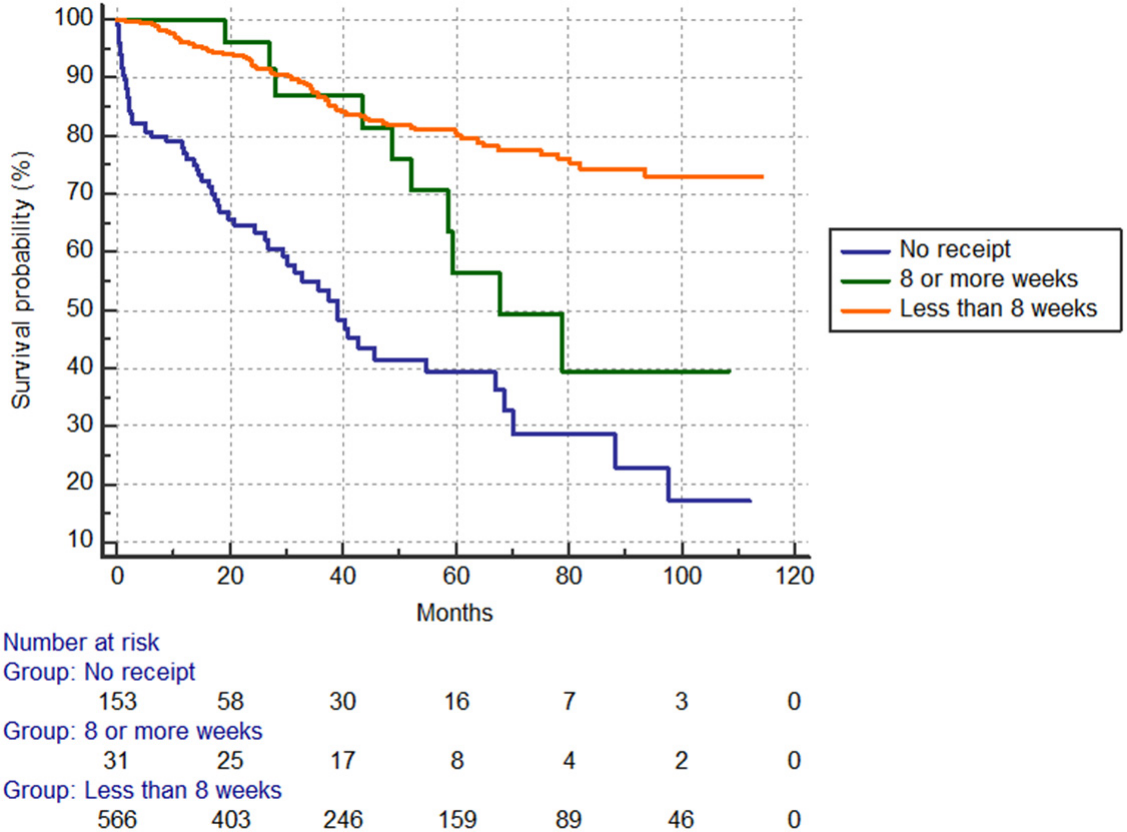
Overall survival according to use of adjuvant chemotherapy use. Five-year rates by group: 39.4% no chemotherapy; 56.5% delay ≥8 weeks; 80.1% <8 weeks (p<0.001).

**Fig 2:**
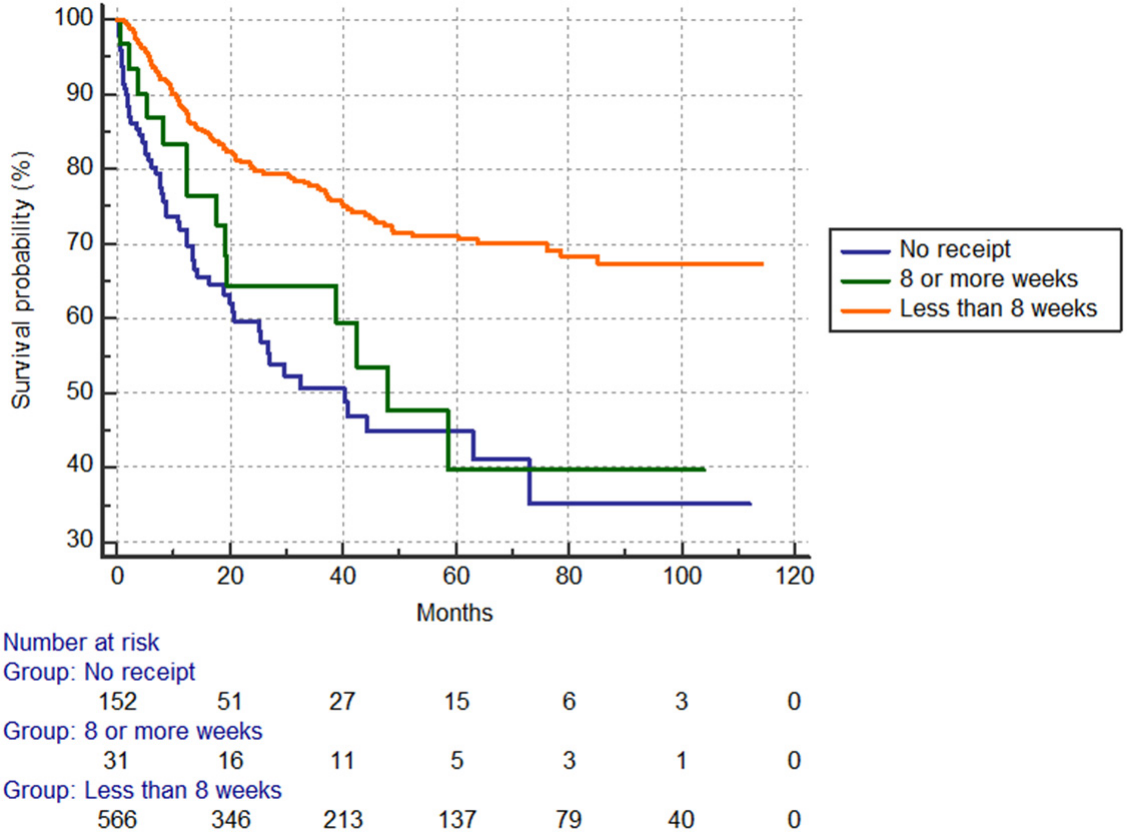
Recurrence-free survival according to use of adjuvant chemotherapy use. Five-year rates by group: 44.8% no chemotherapy; 39.6% delay ≥8 weeks; 71.2% <8 weeks (p<0.001).

The 5-year overall survival rate was 39.4% in the no-chemotherapy group, 56.5% in the ≥8-week group and 80.1% in <8-week group (p<0.001) (**Fig 1**). The 5-year recurrence-free survival rate was 44.8% in the no-chemotherapy group, 39.6% in the ≥8-week delay group and 71.2% in the <8-week group (p<0.001) (**Fig 2**).

### Prognostic survival factors by Cox proportional hazard modeling

Independent prognostic factors for overall survival included TNM III stage (HR = 2.04), chemotherapy use (after ≥8 week: HR = 0.39 and within <8 weeks: HR = 0.22). Independent prognostic factors for recurrence-free survival were TNM III stage (HR = 2.26) and chemotherapy use (within <8 weeks, HR = 0.35) (**Table 4**).

**Table 4:** Prognostic factors for survival on Cox proportional hazard modeling.

	Overall survival		Recurrence-free survival	
	HR (95% CI)	P	HR (95% CI)	P
Use of and time to chemotherapy				
(No use)	Reference		Reference	
(≥8 weeks)	0.39 (0.2–0.78)	0.008	0.73 (0.4–1.33)	0.301
(<8 weeks)	0.22 (0.15–0.32)	<0.001	0.35 (0.24–0.5)	<0.001
TNM (III *vs*. II)	2.04 (1.44–2.9)	<0.001	2.26 (1.65–3.09)	<0.001
Age (≥80 *vs*. <80 years)	1.38 (0.9–2.12)	0.142	0.97 (0.64–1.48)	0.896
30-day complications (+ *vs*.-)	1.29 (0.92–1.79)	0.139	1.18 (0.87–1.59)	0.280
ASA score (3,4 *vs*. 1,2)	1.24 (0.83–1.85)	0.287	1.11 (0.76–1.61)	0.605

HR, hazard ratio; CI, confidence interval; TNM, tumor-node-metastasis; ASA, American Society of Anesthesiologists

## Discussion

The major findings of this study were that different factors affected lack of chemotherapy (older age, ASA score, underlying cerebrovascular disease, stage II disease, postoperative complications, and ICU admission) and delay of chemotherapy (male, rectal cancer, and postoperative complications), with the exception of postoperative complications. Both lack of chemotherapy and delay ≥8 weeks negatively influenced overall and recurrence-free survival. Timely initiation of chemotherapy (<8 weeks) was a favorable prognostic factor for overall and recurrence-free survival.

### Factors associated with the use of adjuvant chemotherapy

Various factors were associated with the use of adjuvant chemotherapy. Older patients receive adjuvant chemotherapy less often than younger patients [12, 13]. Dobie *et al*.[14] found that the rate of adjuvant chemotherapy decreased with advanced age; in their study, 78.9% of patients in the 66- to 70-year-old group received chemotherapy compared to 71.2% in the 71- to 75-year-old group, 56.3% in the 76- to 80-year-old group, 29.8% in the 81- to 85-year-old group and 8.1% in the 86-year-or-older group. We observed the same pattern of decline in chemotherapy use with increasing age.

Patient comorbidity is also related to chemotherapy use. Most studies that measure this outcome use the Charlson comorbidity index, with a higher index score associated with a lower rate of chemotherapy use [13, 15]. Gross *et al*.[16] demonstrated that chronic illnesses such as congestive heart failure, chronic obstructive pulmonary disease, and diabetes influence decreased use of adjuvant chemotherapy. In this study, underlying cerebrovascular disease was a negative predictor for chemotherapy use.

Patient functional status is another important factor when considering chemotherapy use. Age, comorbidity, and functional status are closely related to preoperative health status. Although data are scarce on functional status and use of chemotherapy in an adjuvant setting, patients with stage IV disease and diminished performance status are less likely to receive palliative chemotherapy [17]. As a measure of functional status, we used ASA score, which is a widely adopted grading system for predicting postoperative morbidity and mortality. We observed that ASA scores of 3 and 4 were independent negative predictors for use of chemotherapy.

Tumor histopathology is related to chemotherapy use. Greater numbers of metastatic lymph nodes are associated with more frequent use of chemotherapy[13], likely due to increased consultation needs[18]. Surgical and medical oncologists tend to be more active in recommending chemotherapy in patients with higher-risk tumors. In our study, the number of metastatic nodes was not related to use of chemotherapy, but stage III disease was associated with more frequent use of chemotherapy compared to stage II.

Postoperative complications result in prolonged patient recovery, longer hospital stays, and a higher likelihood of readmission [19, 20]. Accordingly, the presence of postoperative complications is associated with less frequent use of chemotherapy, [21] as was confirmed in this study.

Hospital or physician factors may also affect chemotherapy use. This study was performed in a single tertiary referral center, which allowed good control of systemic factors. In our hospital, adjuvant treatment plans were based on final pathology reports and decided after surgery in weekly meetings of multidisciplinary teams. The treating oncologists explained the overall adjuvant treatment plan and estimated the prognosis of the patient. Patients and family then chose whether or not to receive adjuvant chemotherapy and some refused further treatment. We were unable to evaluate the frequency of patient refusal. In addition, socioeconomic status and race/ethnicity, marital status, area of residence, and income influence the use of chemotherapy [5, 12, 22, 23].

### Factors associated with delay of adjuvant chemotherapy

Older age, black race, prolonged postoperative recovery, severe comorbidity, advanced histologic grade, and unmarried status are associated with delay of adjuvant chemotherapy [19, 22, 24]. In this study, presence of postoperative complications was an independent predictor for chemotherapy delay. In the delayed-chemotherapy group, 90.3% were men (*vs*. 62.5% in the nondelayed group), 83.9% had rectal cancer (*vs*. 43.6% in the nondelayed group), 58.1% experienced postoperative complications (*vs*. 26.9% in the nondelayed group) and 25.8% had anastomotic leakage (*vs*. 2.8% in the nondelayed group). These factors appeared to be related to surgical outcomes. Male sex is a well-known risk factor for anastomotic leakage after rectal cancer surgery [25]. Thus, surgeons should be aware of the detrimental effect of postoperative complications on time to chemotherapy initiation.

Institutional delay between interdepartmental consultations has been suggested as another factor related to chemotherapy delay [6]. In this study, all included patients underwent surgery in our hospital and were discussed at multidisciplinary team meetings. Accordingly, the effect of institutional delay was likely to be small.

### Oncologic outcomes related to use and delay of chemotherapy

Adjuvant chemotherapy improves overall and recurrence-free rates of survival in stage II and III colorectal cancer[12]. One meta-analysis showed that more than 8 weeks of delay to adjuvant chemotherapy in stage III colorectal cancer worsened overall survival [22]. However, Zeig-Owens *et al*.[23] demonstrated that a chemotherapy delay of more than 45 days was not associated with survival of patients with stage II and III colon cancer. Additional controversies are over the definition of delay, which has been defined as 45 days[23, 26], 8 weeks[27–29], and 3 months[19] in various studies. In this study, delay of chemotherapy ≥8 weeks showed oncologic benefits in terms of overall survival when compared to no chemotherapy, but no benefits in recurrence-free survival. Timely initiation of chemotherapy in <8 weeks was a favorable prognostic factor for overall and recurrence-free survival. Postoperative complications are suggested to be risk factors for inferior oncologic outcomes[30]. In this study, surgical complications were associated with lack of and delay of chemotherapy, but were not a prognostic factor. This study used 8 weeks as the cutoff for chemotherapy delay. This decision was based on Health Insurance Review Assessment Service of South Korea claims data on time to chemotherapy initiation in patients with colorectal cancer. In Korea, if patients do not receive adjuvant chemotherapy or undergo chemotherapy after more than 8 weeks, treating physicians must notify a government agency.

This study was limited by its single-center, retrospective design. In addition, data regarding chemotherapy dose reduction or discontinuation were not available. Although all medical records were reviewed, we acknowledge that some treatment-related events such as ICU admission or transfusion could have been missed.

In summary, we identified factors associated with not receiving chemotherapy or experiencing a ≥8-week delay to chemotherapy in an adjuvant setting after surgery for stage II or III colorectal cancer. Among the factors investigated, the presence of postoperative complications correlated with both not receiving and delay of chemotherapy. Timely initiation of chemotherapy (<8 weeks postoperative) was a favorable prognostic factor for overall and recurrence-free survival. To increase the proportion of patients receiving chemotherapy and timely initiation of chemotherapy, surgical complications should be minimized after curative resection.

## References

[1] Andre T, Boni C, Navarro M, Tabernero J, Hickish T, Topham C, et al Improved overall survival with oxaliplatin, fluorouracil, and leucovorin as adjuvant treatment in stage II or III colon cancer in the MOSAIC trial. Journal of clinical oncology: official journal of the American Society of Clinical Oncology. 2009;27(19):3109–16. Epub 2009/05/20. 10.1200/jco.2008.20.6771 . 19451431

[2] Gray R, Barnwell J, McConkey C, Hills RK, Williams NS, Kerr DJ. Adjuvant chemotherapy versus observation in patients with colorectal cancer: a randomised study. Lancet. 2007;370(9604):2020–9. Epub 2007/12/18. 10.1016/s0140-6736(07)61866-2 . 18083404

[3] Dube S, Heyen F, Jenicek M. Adjuvant chemotherapy in colorectal carcinoma: results of a meta-analysis. Diseases of the colon and rectum. 1997;40(1):35–41. Epub 1997/01/01. . 9102259 10.1007/BF02055679

[4] DeSantis CE, Lin CC, Mariotto AB, Siegel RL, Stein KD, Kramer JL, et al Cancer treatment and survivorship statistics, 2014. CA: a cancer journal for clinicians. 2014;64(4):252–71. 10.3322/caac.21235 . 24890451

[5] Etzioni DA, El-Khoueiry AB, Beart RW Jr. Rates and predictors of chemotherapy use for stage III colon cancer: a systematic review. Cancer. 2008;113(12):3279–89. Epub 2008/10/28. 10.1002/cncr.23958 . 18951522

[6] Biagi JJ, Raphael MJ, Mackillop WJ, Kong W, King WD, Booth CM. Association between time to initiation of adjuvant chemotherapy and survival in colorectal cancer: a systematic review and meta-analysis. JAMA: the journal of the American Medical Association. 2011;305(22):2335–42. Epub 2011/06/07. 10.1001/jama.2011.749 . 21642686

[7] National Comprehensive Cancer Network. Rectal cancer (Version 1.2015) [cited 2015 January 7]. Available from: http://www.nccn.org/professionals/physician_gls/pdf/rectal.pdf.

[8] National Comprehensive Cancer Network. Colon cancer (Version 2.2015) [cited 2015 January 7]. Available from: http://www.nccn.org/professionals/physician_gls/pdf/colon.pdf.

[9] Taal BG, Van Tinteren H, Zoetmulder FA. Adjuvant 5FU plus levamisole in colonic or rectal cancer: improved survival in stage II and III. British journal of cancer. 2001;85(10):1437–43. Epub 2001/11/27. 10.1054/bjoc.2001.2117 ; PubMed Central PMCID: PMCPmc2363941. 11720425 PMC2363941

[10] Wolmark N, Rockette H, Fisher B, Wickerham DL, Redmond C, Fisher ER, et al The benefit of leucovorin-modulated fluorouracil as postoperative adjuvant therapy for primary colon cancer: results from National Surgical Adjuvant Breast and Bowel Project protocol C-03. Journal of clinical oncology: official journal of the American Society of Clinical Oncology. 1993;11(10):1879–87. Epub 1993/10/01. . 8410113 10.1200/JCO.1993.11.10.1879

[11] von Elm E, Altman DG, Egger M, Pocock SJ, Gotzsche PC, Vandenbroucke JP. The Strengthening the Reporting of Observational Studies in Epidemiology (STROBE) statement: guidelines for reporting observational studies. Lancet. 2007;370(9596):1453–7. Epub 2007/12/08. 10.1016/s0140-6736(07)61602-x . 18064739

[12] Ayanian JZ, Zaslavsky AM, Fuchs CS, Guadagnoli E, Creech CM, Cress RD, et al Use of adjuvant chemotherapy and radiation therapy for colorectal cancer in a population-based cohort. Journal of clinical oncology: official journal of the American Society of Clinical Oncology. 2003;21(7):1293–300. Epub 2003/03/29. . 12663717 10.1200/JCO.2003.06.178

[13] Schrag D, Cramer LD, Bach PB, Begg CB. Age and adjuvant chemotherapy use after surgery for stage III colon cancer. Journal of the National Cancer Institute. 2001;93(11):850–7. Epub 2001/06/08. . 11390534 10.1093/jnci/93.11.850

[14] Dobie SA, Baldwin LM, Dominitz JA, Matthews B, Billingsley K, Barlow W. Completion of therapy by Medicare patients with stage III colon cancer. Journal of the National Cancer Institute. 2006;98(9):610–9. Epub 2006/05/04. 10.1093/jnci/djj159 ; PubMed Central PMCID: PMCPmc3124351. 16670386 PMC3124351

[15] McGory ML, Zingmond DS, Sekeris E, Bastani R, Ko CY. A patient's race/ethnicity does not explain the underuse of appropriate adjuvant therapy in colorectal cancer. Diseases of the colon and rectum. 2006;49(3):319–29. Epub 2006/02/14. 10.1007/s10350-005-0283-6 . 16475031

[16] Gross CP, McAvay GJ, Guo Z, Tinetti ME. The impact of chronic illnesses on the use and effectiveness of adjuvant chemotherapy for colon cancer. Cancer. 2007;109(12):2410–9. Epub 2007/05/19. 10.1002/cncr.22726 . 17510973

[17] Kim YW, Kim IY. The Role of Surgery for Asymptomatic Primary Tumors in Unresectable Stage IV Colorectal Cancer. Ann Coloproctol. 2013;29(2):44–54. Epub 2013/05/24. 10.3393/ac.2013.29.2.44 PubMed Central PMCID: PMCPmc3659242. 23700570 PMC3659242

[18] Luo R, Giordano SH, Freeman JL, Zhang D, Goodwin JS. Referral to medical oncology: a crucial step in the treatment of older patients with stage III colon cancer. The oncologist. 2006;11(9):1025–33. Epub 2006/10/13. 10.1634/theoncologist.11-9-1025 ; PubMed Central PMCID: PMCPmc1913211. 17030645 PMC1913211

[19] Cheung WY, Neville BA, Earle CC. Etiology of delays in the initiation of adjuvant chemotherapy and their impact on outcomes for Stage II and III rectal cancer. Diseases of the colon and rectum. 2009;52(6):1054–63; discussion 64. 10.1007/DCR.0b013e3181a51173 . 19581846

[20] Hendren S, Birkmeyer JD, Yin H, Banerjee M, Sonnenday C, Morris AM. Surgical complications are associated with omission of chemotherapy for stage III colorectal cancer. Diseases of the colon and rectum. 2010;53(12):1587–93. 10.1007/DCR.0b013e3181f2f202 . 21178851

[21] Baldwin LM, Dobie SA, Billingsley K, Cai Y, Wright GE, Dominitz JA, et al Explaining black-white differences in receipt of recommended colon cancer treatment. Journal of the National Cancer Institute. 2005;97(16):1211–20. Epub 2005/08/18. 10.1093/jnci/dji241 ; PubMed Central PMCID: PMCPmc3138542. 16106026 PMC3138542

[22] Des Guetz G, Nicolas P, Perret GY, Morere JF, Uzzan B. Does delaying adjuvant chemotherapy after curative surgery for colorectal cancer impair survival? A meta-analysis. European journal of cancer (Oxford, England: 1990). 2010;46(6):1049–55. Epub 2010/02/09. 10.1016/j.ejca.2010.01.020 . 20138505

[23] Zeig-Owens R, Gershman ST, Knowlton R, Jacobson JS. Survival and time interval from surgery to start of chemotherapy among colon cancer patients. Journal of registry management. 2009;36(2):30–41; quiz 61–2. Epub 2009/08/22. . 19694115

[24] Hershman D, Hall MJ, Wang X, Jacobson JS, McBride R, Grann VR, et al Timing of adjuvant chemotherapy initiation after surgery for stage III colon cancer. Cancer. 2006;107(11):2581–8. Epub 2006/11/02. 10.1002/cncr.22316 . 17078055

[25] Law WI, Chu KW, Ho JW, Chan CW. Risk factors for anastomotic leakage after low anterior resection with total mesorectal excision. Am J Surg. 2000;179(2):92–6. Epub 2000/04/25. . 10773140 10.1016/s0002-9610(00)00252-x

[26] Bayraktar UD, Chen E, Bayraktar S, Sands LR, Marchetti F, Montero AJ, et al Does delay of adjuvant chemotherapy impact survival in patients with resected stage II and III colon adenocarcinoma? Cancer. 2011;117(11):2364–70. Epub 2011/06/01. 10.1002/cncr.25720 . 24048783

[27] Ahmed S, Ahmad I, Zhu T, Arnold FP, Faiz Anan G, Sami A, et al Early discontinuation but not the timing of adjuvant therapy affects survival of patients with high-risk colorectal cancer: a population-based study. Diseases of the colon and rectum. 2010;53(10):1432–8. Epub 2010/09/18. 10.1007/DCR.0b013e3181e78815 . 20847626

[28] Chau I, Norman AR, Cunningham D, Tait D, Ross PJ, Iveson T, et al A randomised comparison between 6 months of bolus fluorouracil/leucovorin and 12 weeks of protracted venous infusion fluorouracil as adjuvant treatment in colorectal cancer. Annals of oncology: official journal of the European Society for Medical Oncology / ESMO. 2005;16(4):549–57. Epub 2005/02/08. 10.1093/annonc/mdi116 . 15695501

[29] Czaykowski PM, Gill S, Kennecke HF, Gordon VL, Turner D. Adjuvant chemotherapy for stage III colon cancer: does timing matter? Diseases of the colon and rectum. 2011;54(9):1082–9. Epub 2011/08/10. 10.1097/DCR.0b013e318223c3d6 . 21825887

[30] Law WL, Choi HK, Lee YM, Ho JW. The impact of postoperative complications on long-term outcomes following curative resection for colorectal cancer. Annals of surgical oncology. 2007;14(9):2559–66. Epub 2007/05/25. 10.1245/s10434-007-9434-4 . 17522945

